# Structural basis for the evolution of cyclic phosphodiesterase activity in the U6 snRNA exoribonuclease Usb1

**DOI:** 10.1093/nar/gkz1177

**Published:** 2019-12-13

**Authors:** Yuichiro Nomura, Eric J Montemayor, Johanna M Virta, Samuel M Hayes, Samuel E Butcher

**Affiliations:** Department of Biochemistry, University of Wisconsin, Madison, WI 53706, USA

## Abstract

U6 snRNA undergoes post-transcriptional 3′ end modification prior to incorporation into the active site of spliceosomes. The responsible exoribonuclease is Usb1, which removes nucleotides from the 3′ end of U6 and, in humans, leaves a 2′,3′ cyclic phosphate that is recognized by the Lsm2–8 complex. *Saccharomycescerevisiae* Usb1 has additional 2′,3′ cyclic phosphodiesterase (CPDase) activity, which converts the cyclic phosphate into a 3′ phosphate group. Here we investigate the molecular basis for the evolution of Usb1 CPDase activity. We examine the structure and function of Usb1 from *Kluyveromyces marxianus*, which shares 25 and 19% sequence identity to the *S. cerevisiae* and *Homo sapiens* orthologs of Usb1, respectively. We show that *K. marxianus* Usb1 enzyme has CPDase activity and determined its structure, free and bound to the substrate analog uridine 5′-monophosphate. We find that the origin of CPDase activity is related to a loop structure that is conserved in yeast and forms a distinct penultimate (*n* – 1) nucleotide binding site. These data provide structural and mechanistic insight into the evolutionary divergence of Usb1 catalysis.

## INTRODUCTION

Usb1 is a member of the 2H phosphodiesterase superfamily of enzymes, which contain two vicinal active site HxS/T motifs that are essential for catalysis. The 2H superfamily can be sub-divided into HxT and HxS enzymes, the latter of which contains Usb1. 2H superfamily enzymes act on myriad RNAs and nucleotide substrates, and are capable of many catalytic activities, including 2′,5′ RNA ligase or nuclease, 2′,5′ or 3′,5′-phosphodiesterase, and 1″,2″-cyclic or 2′,3′-cyclic phosphodiesterase (CPDase) activities (1). These divergent activities are all thought to utilize two catalytic histidines within the central HxS/T motifs that act as a general acid and base, while the serine or threonine residues help coordinate substrates and assist in transition state stabilization (2–5). Although the overall sequence conservation among 2H enzymes is rather low, all crystal structures of family members determined thus far display a characteristic fold with conserved terminal and transit lobes and the HxS/T motifs centrally positioned in a substrate binding cleft (3,5–15).

**Figure 1.**
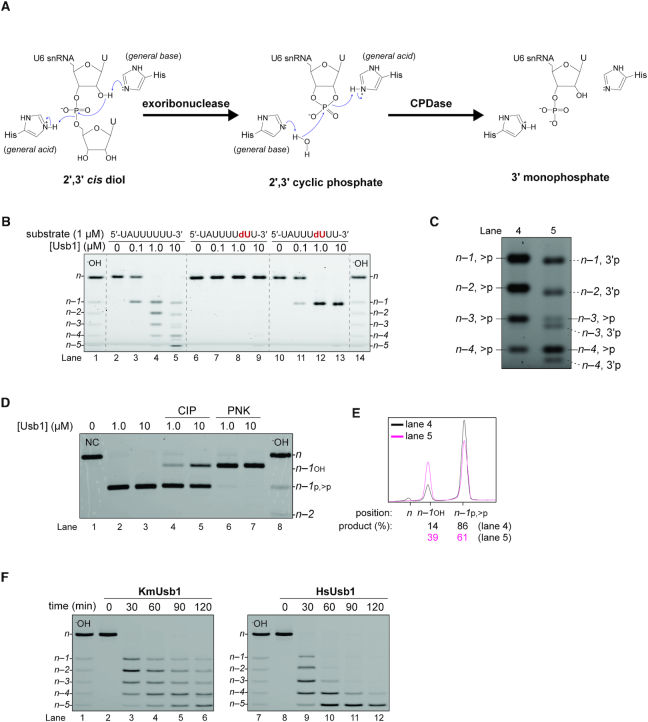
*In vitro* characterization of *K. marxianus* Usb1 activity. (**A**) Schematic cartoon of the catalytic mechanism of Usb1. Usb1 is a 3′-5′ exoribonuclease that leaves a 2′,3′-cyclic phosphate. Some orthologs of Usb1 have additional CPDase activity that hydrolyzes the cyclic phosphodiester linkage to yield a 3′-monophosphate group. (**B**) The exoribonuclease activity of *K. marxianu*s Usb1. Use of a minimal substrate shows that KmUsb1 is indeed an exoribonuclease (lanes 2–5). The ribonuclease activity is completely blocked by 2′-deoxy modification of the *n* – 1 uridine (lanes 6–9). 2′-Deoxy modification of the *n* – 2 uridine results in single cleavage products (lanes 10–13) that allow facile monitoring of enzymatic activity. (**C**) Close-up view of products in lanes 4 and 5 of Figure 1B. Cleavage products with two types of 3′-end phosphates are observed at higher concentrations of *K. marxianus* enzyme for substrate. (**D**) 3′-end phosphoryl formation by Usb1. A single-cleavage substrate (5′-UAUUUdUUU-3′) was incubated with 0–10 μM KmUsb1 and products (*n* – 1_P_, lanes 2 and 3) were then treated with CIP (lanes 4 and 5) or PNK (lanes 6 and 7). The shifted band (*n* – 1_OH_) is generated by removal of 3′-end phosphoryl groups by either CIP or PNK, the former of which can only remove non-cyclic phosphates (**E**) Comparison of products in the presence of 1.0 and 10 μM enzyme. Product (%) in lanes 4 and 5 shown in Figure 1D was quantified upon fluorescence intensity and indicates the relative CPDase activity. (**F**) Comparison of *K. marxianus* and human Usb1 activities. Reaction mixtures contained 1 μM unmodified substrate (UAUUUUUU) and either 10 μM KmUsb1 (lanes 2–6) or HsUsb1 (lanes 8–12).

Usb1 was first identified in humans as being the mutated gene responsible for the genetic disorder poikiloderma with neutropenia (16) and was soon thereafter identified as the 3′-5′-exoribonuclease responsible for 3′-end maturation of U6 snRNA (17,18). In *Saccharomycescerevisiae*, Usb1 is an essential enzyme (17). *In vitro* analyses showed that human Usb1 (hereafter, HsUsb1) has distributive 3′-5′-phosphodiesterase activity that progressively shortens the U6 3′-tail, leaving products with 2′,3′-cyclic phosphates (5,11,14,18,19). In contrast, *S. cerevisiae* Usb1 (hereafter, ScUsb1) predominantly removes a single nucleotide from U6, leaving a 3′ monophosphate which strongly inhibits further processing (Figure 1A) (14). Thus, ScUsb1 has 2′-CPDase activity that hydrolyzes the 2′,3′-cyclic phosphate product into a 3′ monophosphate, an activity that is not present in the human enzyme.

Several Usb1 crystal structures have been determined from human and *S*. *cerevisiae* (5,11,14). Despite sharing low sequence identity (<20%), the human and *S*. *cerevisiae* Usb1 enzymes have overall highly similar structures. Structures of HsUsb1 bound to nucleotides and intact RNA, in combination with kinetic analyses and QM/MM simulations, have revealed insights into its catalytic mechanism (5,14). However, the mechansim of CPDase activity in the *S. cerevisiae* enzyme remains less well understood. No structures of ScUsb1 bound to nucleotides or RNA have been determined, nor are there any known structures for a 2H superfamily enzyme with 2′-CPDase activity bound to substrate. It is also not known if ScUsb1 is unique, or if other orthologs of Usb1 harbor CPDase activity. The presence of active site HxS motifs alone cannot explain CPDase activity, as these regions in ScUsb1 and HsUsb1 are superimposable within 0.4 Å r.m.s.d. for all 32 atoms of the histidines and serines in the HxS motifs (14). Thus, the molecular basis for 2′-CPDase activity and the divergent activities of Usb1 enzymes cannot be explained by existing structural data.

U6 snRNA is a central active site component of the spliceosome, a large macromolecular machine that catalyzes precursor messenger RNA splicing (20,21). Nascent U6 transcripts, which are synthesized by RNA polymerase III, have heterogeneous polyuridine tails with 2′,3′-*cis*-diols (22,23). Processing by Usb1 facilitates the recruitment of U6 to the spliceosome by increasing the binding affinity of the Lsm2–8 complex (14,24,25). In particular, the C-terminus of the Lsm8 protein interacts with the 3′ end of U6. We previously reported that the C-terminal sequences of Lsm8 from various organisms fall into two families, and hypothesized that they evolved to recognize cyclic phosphates and 3′ phosphates (25). The Lsm8 proteins from *S*. *cerevisiae* and related yeast have an extended lysine rich C-terminal sequence, which interacts electrostatically with the negatively charged 3′ phosphate group. In constrast, the Lsm8 proteins that recognize a cyclic phosphate lack this C-terminal extension. We therefore further hypothesized that Lsm8 sequence alignments can be used to predict U6 3′ chemistry, and by extension Usb1 activity, *in vivo*. For example, alignment of the putative Lsm8 ortholog from the ascomycetous yeast *Kluyveromyces marxianus* shows its C-terminus is similar to *S. cerevisiae*, suggesting the presence of a 3′ phosphate on its U6 snRNA and therefore CPDase activity in the corresponding ortholog of Usb1 (hereafter, KmUsb1) (Supplementary Figure S1).

Molecular clock measurements estimate that humans and yeast diverged from a common ancestor approximately 1 billion years ago, whereas *K. marxianus* and *S. cerevisiae* shared a common ancestor 10^8^ years ago (26). In order to provide further information on the structure and divergent catalytic mechanisms of Usb1 orthologs, we have characterized the *in vitro* activity and structure of the KmUsb1 enzyme. Our *in vitro* activity assays show that KmUsb1 shares properties of both ScUsb1 and HsUsb1. For example, KmUsb1 displays distributive 3′-5′-exoribonuclease processing that can progressively shorten the U6 snRNA 3′ end, which is similar to the human enzyme but not ScUsb1. On the other hand, the enzyme has measurable CPDase activity similar to ScUsb1 but not HsUsb1. In all three orthologs, Usb1 is strongly inhibited by a terminal 3′ phosphate.

We report crystal structures of KmUsb1, both free and bound to the substrate analog uridine 5′-monophosphate. The overall architecture is similar to the human and *S. cerevisiae* enzymes, and the substrate analog occupies the terminal nucleotide (‘*n*’) binding pocket in a manner that is very similar to HsUsb1. Structural comparisons and activity assays lead us to hypothesize that the origin of CPDase activity is related to the *n–1* nucleotide binding site, for which both yeast species possess a similar loop architecture that is absent in the human enzyme.

## MATERIALS AND METHODS

### Sample preparation

The DNA sequence coding for full-length *K. marxianus* Usb1 was codon optimized and synthesized by Integrated DNA Technologies for heterologous overexpression in *Escherichia coli* (Supplementary Data). The Usb1 open reading frame was cloned into the NdeI and BamHI sites of pET3a plasmid carrying the coding sequence of an octahistidine tag, maltose binding protein and TEV protease cleavage site upstream of the NdeI site (27,28). The crystallizable N-terminal deletion mutant (lacking the first 58 residues) was also constructed in the same way. Alanine substitution mutation was introduced by inverse PCR with primers designed for the target DNA sequence. All constructs were expressed and purified by the following protocol. *Escherichia coli* competent cells, BL21(DE3)pLysS (Invitrogen) were transformed with the above plasmids and protein expression was induced at an OD_600_ of ∼2–3 by addition of 1 mM IPTG, then grown at 16°C overnight in terrific broth medium supplemented with 1% glycerol, 100 μg/ml of ampicillin and 30 μg/ml of chloramphenicol. Cells were harvested by centrifugation and resuspended in immobilized metal affinity chromatography (IMAC) buffer (50 mM HEPES, pH 7.4, 500 mM NaCl, 25 mM imidazole, 1 mM TCEP–HCl and 10% glycerol) supplemented with DNase I, lysozyme and protease inhibitors, then lysed by sonication. The soluble fraction obtained by centrifugation was purified via Ni-NTA agarose resin (QIAGEN), and then eluted with IMAC buffer containing 500 mM imidazole. The eluate was dialyzed against IEX buffer (20 mM HEPES, pH 7.4, 100 mM NaCl, 1 mM TCEP–HCl and 10% glycerol) supplemented with ∼1 mg of TEV protease at 4°C overnight. Subsequent purification was performed with amylose resin (New England Biolabs) for removal of maltose binding protein. The resultant fraction was directly applied to a HiTrap Heparin-HP column (GE Healthcare) and eluted with a linear gradient from 50 to 600 mM NaCl in IEX buffer. For crystallization, the final product was further dialyzed against buffer (100 mM sodium phosphate, pH 6.8, 100 mM NaCl and 1 mM TCEP–HCl), concentrated to 24 mg/ml with Amicon 10 kDa spin filters (Millipore), and then stored at –80°C until use.

### Crystallization

Crystallization screening for N-terminally truncated *K. marxianus* Usb1 was performed by vapor diffusion in sitting drops at 4°C using commercial crystal screens (JCSG-plus, MIDAS, Morpheus, PACT premier from Molecular Dimensions, Index HT, PEGRx HT from Hampton Research, and the Cryos Suite from Qiagen). In two months, crystals were found in 1 M potassium sodium tartrate, 100 mM HEPES, pH 7.4 and 30% glycerol. This condition was subsequently optimized by hanging-drop vapor diffusion with 1 μl protein and 1 μl reservoir solution (1.2 M potassium sodium tartrate, 100 mM HEPES, pH 7.5, 21% glycerol) at 4°C for 1 week. Initial phases could not be inferred by molecular replacement with either of the known human or *S*. *cerevisiae* Usb1 structures. Phase determination was therefore accomplished by single isomorphous replacement with anomalous scattering using a uranyl acetate derivative (29). In order to prepare the heavy atom derivative, the above crystals were transferred into a fresh drop (1.2 M potassium sodium tartrate, 100 mM HEPES, pH 7.5, 21% glycerol, 100 mM NaCl and 1 mM TCEP–HCl) saturated with uranyl acetate, and allowed to incubate at 4°C for two days. Nine uranium atoms were estimated to reside in the asymmetric unit based on clear density in an anomalous difference map. For the native structure, crystals were generated with 1 μl protein and 1μl reservoir solution (1.2 M potassium sodium tartrate, 100 mM HEPES, pH 7.5, 30% glycerol). Several glycerol molecules are observed at the active site of the native structure, which likely prevented generating a co-crystal structure of KmUsb1 bound to uridine 5′-monophosphate (5′-UMP) (Supplementary Figure S2A and B). The subsequent trials were thus performed by a modified soaking method in which the native crystals were transferred into a fresh drop containing 1.2 M potassium sodium tartrate, 100 mM HEPES, pH 7.5, 30% PEG 400, 10 mM 5′-UMP and incubated at 4°C overnight. Many partial fragments of PEG 400, instead of glycerol molecules, were visible in the final structure, but do not interfere with accommodation of 5′-UMP in the active site.

### Data collection and refinement

Diffraction data were collected at 100 K on beamline 24-ID-E or 21-ID-F of the Advanced Photon Source using a PILATUS 6MF or Rayonix MX300 detector, respectively. Data were integrated, indexed and scaled with XDS (30) and AIMLESS (31). The initial phases were determined using SHELXC/D/E pipeline (32) as implemented in HKL2Map (Supplementary Figure S3) (33). Automated model building was accomplished in RESOLVE and PHENIX (34,35). Initial phases for the native structure were determined by molecular replacement using the best model from the uranium bound structure. The diffraction data for the UMP bound crystals exhibited anisotropy, and were therefore subjected to ellipsoidal truncation and scaling by the STARANISO server (36). The subsequent refinement was performed via iterative rounds of manual model building in COOT (37) and automated refinement with individual isotropic B-factors, TLS and NCS restraints in PHENIX and REFMAC (38,39). Ligand occupancies were refined in PHENIX. Simulated annealing omit maps were generated in PHENIX, and used for final appraisal of the bound ligands. All figures were generated with PyMOL (http://www.pymol.org/).

### Biochemical assays

*K. marxianus* U6 nucleotides 104–110 with an additional nucleotide at 3′ end were utilized as substrate RNAs for *K. marxianus* Usb1. The sequence of *K. marxianus* U6 was inferred from homology to the close U6 homologs in *Kluyveromyces lactis* and *S*. *cerevisiae*, with 94 and 89% sequence identity, respectively. The RNA was labeled with 6-carboxyfluorescein at the 5′ end (5′-FAM) and modified with four different nucleotides at the 3′ end to observe the nucleotide specificity. In order to ensure single cleavage of these substrates, 2′-deoxyuridine was incorporated at the antepenultimate position. All substrate RNAs were purchased from Integrated DNA Technologies (see Supplementary Data) and were gel purified prior to usage.

The assay conditions were first optimized at a wide range of pH values (4.0–10.0) using a buffer containing 4 mM each of acetate, bis-tris, HEPES, Tris and CHES. Assays were performed at 37°C for 30 min using an equal concentration of substrate RNA and enzyme (Supplementary Figure S4). Unless stated otherwise, the following assays were generally conducted at 37°C for 30 min with an optimized buffer (20 mM bis–tris, pH 6.5, 100 mM NaCl, 1 mM EDTA, 1 mM TCEP–HCl) containing 1 μM RNA and 0–10 μM Usb1. To determine the 3′-end phosphate modification left by Usb1, reaction products were treated with 10 units of CIP or PNK under optimum buffer conditions as indicated by the manufacturer (New England Biolabs). To compare products shortened by Usb1 orthologs, 1 μM RNA (FAM-UAUUUUUU) was incubated with either 10 μM human or *K. marxianus* enzymes at 37°C for the indicated times (5).

The independent exoribonuclease activity was monitored in the same conditions as above with a slight modification. Briefly, 1 μM RNA was incubated with 1 μM Usb1 variants at 37°C for 10 min, followed by 3′-end dephosphorylation using either calf-intestinal alkaline phosphatase (CIP) or T4 polynucleotide kinase (PNK) to confirm that CPDase activity is undetectable. To prepare substrate for monitoring CPDase activity, 50 μM RNA (FAM-UAUUUdUUA) was incubated with 5 μM HsUsb1 at 37°C for 1 h with an optimized buffer (5). The single-cleavage product (FAM-UAUUUdUU>p) was isolated by denaturing gel extraction and purified with HiTrap Q column (GE Healthcare). The quality of resulting product was confirmed by CIP and PNK treatment (Supplementary Figure S5). The CPDase activity was observed in an optimized buffer containing 1 μM substrate and 20 μM Usb1 variants at 37°C for 1 hour, followed by 3′-end dephosphorylation using CIP.

The marker RNA (^–^OH) was generated by partial hydrolysis of unmodified RNA (FAM-UAUUUUUU) at 95°C for 10 min in 50 mM sodium carbonate (pH 9.2) and 1 mM EDTA. Reaction progress was determined by denaturing electrophoresis on 20% (19:1) polyacrylamide gels containing 8 M urea, 100 mM tris-borate and 1 mM EDTA, with subsequent visualization of fluorescent substrates and products on a Typhoon FLA 9000 (GE Healthcare).

## RESULTS

### Characterization of *K. marxianus* Usb1 activity

A candidate ortholog of Usb1 in *K. marxianus* was first identified by searching for sequences that harbor two HxS motifs and share similarity with confirmed orthologs of the enzyme. An uncharacterized open reading frame from *K. marxianus* (YLR132C) was identified with low sequence similarity to human and *S*. *cerevisiae* Usb1 (18.9% and 24.7% sequence identity, respectively) (Supplementary Figure S6). We cloned the open reading frame corresponding to this protein and expressed it in *E. coli*. We then used a minimal substrate corresponding to the 3′-end region of *K. marxianus* U6 snRNA to show that the enzyme is indeed an exoribonuclease (Figure 1B). Modified versions of this substrate, with either a penultimate (*n* – 1) or antepenultimate (*n* – 2) 2′-deoxyuridine, show that the KmUsb1 requires an attacking 2′ OH group for the cleavage reaction and that the enzyme lacks endoribonuclease activity, as do other characterized orthologs of Usb1 (Figure 1A and B) (5,14). When 1.0 μM substrate is incubated in the presence of equimolar or ten-fold excess of KmUsb1 enzyme, multiple products of heterogeneous length are observed (Fig. 1B). This behavior is highly similar to the human enzyme, but differs from *S*. *cerevisiae* Usb1 which predominantly removes a single nucleotide (5,14). The human enzyme is catalytically inefficient with a *k*_cat_/*K*_m_ = 10^2^ M^−1^ s^−1^ for a single cleavage event (5). Our measurements of KmUsb1 activity indicate that it is also a catalytically inefficient enzyme *in vitro*. Interestingly, when the enzyme is in excess, the *n–1* and *n–2* products have faster electrophoretic mobility relative to the products obtained with a lower concentration of enzyme, and the *n* – 3 and *n* – 4 products are doublets (Figure 1C, compare lanes 4 and 5). We hypothesized that the faster mobility products are terminal 3′ phosphates, which have more negative charge due to a second ionization constant near pH 7. We tested this hypothesis by reacting the single cleavage substrate (*n* – 2 deoxyuridine) with KmUsb1 and then treating the products with the enzymes CIP and PNK (Figure 1D and E). CIP can hydrolyze terminal but not cyclic phosphates, whereas PNK can hydrolyze both. After half an hour of processing with either 1 or 10 micromolar enzyme, the single cleavage substrate appears to be fully processed (Figure 1D, lanes 2 and 3). However, at 1 micromolar enzyme the products have mostly cyclic phosphates and are resistant to CIP treatment (86% cyclic phosphate versus 14% terminal phosphate; Figure 1E). At higher concentration of enzyme, more CPDase activity is observed as the amount of terminal phosphate products more than double (Figure 1D and E). The observed behavior is consistent with distributive processing in which the exoribonuclease activity is more efficient than the CPDase activity, with the enzyme dissociating after the first exoribonuclease step and then rebinding to hydrolyze the cyclic phosphate product. The presence of measurable CPDase activity is consistent with alignment of Lsm8 sequences (Supplementary Figure S1).

We also compared *K. marxianus* and human Usb1 activities by time-course experiments in which 1 μM unmodified RNA was processed with 10 μM enzyme (Figure 1F). The *n* – 1, *n* – 2 and *n* – 3 products shortened by KmUsb1 are still detectable after two-hour incubation, whereas the corresponding products are unmeasurable in HsUsb1 (Figure 1F, lanes 6 vs. 12). This difference suggests that the products with monophosphates inhibit further exoribonuclease activity of KmUsb1, as observed previously for ScUsb1. Indeed, we find that KmUsb1 is completely inhibited by a 3′ phosphate (Supplementary Figure S7). Thus, inhibition by a 3′ phosphate is a common property of all tested Usb1 orthologs, including HsUsb1 (11), ScUsb1 (14) and KmUsb1. Distributive processing by KmUsb1 is thus diminished by the slow accumulation of inhibitory 3′ phosphate products, which are not produced by HsUsb1.

### Structure of *K. marxianus* Usb1

To determine the structure of KmUsb1, crystallization trials for full-length protein (residues 1–275) were performed using several commercial crystal screens but no crystals could be obtained. Subsequent trials were performed with N-terminally truncated Usb1 by removing the first 58 amino acids (residues 59–275, Supplementary Figure S6) that was designed to mimic the crystallizable *S*. *cerevisiae* and human orthologs of Usb1 (5,11,14). The structure of KmUsb1 was determined by single isomorphous replacement with anomalous scattering (SIRAS) methods (Supplementary Figure S3) and a uranyl acetate heavy atom derivative (Table 1). The asymmetric unit contains three subunits (Supplementary Figure S2A), each of which is essentially identical in structure with an average alpha carbon pairwise r.m.s.d. of 0.63 Å for residues 61–275.

**Table 1.** Data collection and refinement statistics

	KmUsb1 PDB 6PFQ	KmUsb1–5′-UMP PDB 6PGL	KmUsb1–uranyl acetate phasing derivative
**Data collection**			
Wavelength (Å)	0.9792	0.9787	0.9792
Resolution range (Å)	107.36–1.80 (1.84–1.80)	79.52–1.84 (2.04–1.84)	114.77–2.41 (2.50–2.41)
Space group	*P*2_1_2_1_2_1_	*P*2_1_2_1_2_1_	*P*2_1_2_1_2_1_
Unit cell dimensions (Å)	69.93, 107.36, 113.90	69.96, 110.34, 114.70	70.29, 108.17, 114.77
Total reflections	543,553 (30,449)	462,710 (20,748)	460,112 (50,947)
Unique reflections	80,145 (4,528)	56,622 (2,827)	34,579 (3,604)
Multiplicity	6.8 (6.7)	8.2 (7.3)	13.3 (14.1)
Completeness (%)			
Spherical	100 (100)	72.5 (13.5)	100 (100)
Ellipsoidal	NA	94.9 (64.7)	NA
Mean I/σ(I)	16.3 (1.5)	15.3 (1.7)	15.0 (0.9)
Wilson *B* (Å^2^)	24.98	31.98	57.49
*R*_merge_	0.066 (1.254)	0.111 (1.130)	0.101 (2.928)
*R*_pim_	0.027 (0.520)	0.043 (0.480)	0.029 (0.801)
CC_1/2_	0.999 (0.615)	0.999 (0.641)	0.999 (0.479)
Figure of merit			0.56
*R*_work_/*R*_free_	0.19/0.22 (0.30/0.33)	0.19/0.22 (0.47/0.61)	
No. of atoms			
Total	5400	5420	
Macromolecules	4898	4803	
Ligands	66	198	
Water	436	419	
R.m.s.d., bonds	0.011	0.008	
R.m.s.d., angles	1.177	0.987	
Ramachandran favored (%)	100	99.53	
Ramachandran outliers (%)	0	0	
Average *B* factor (Å^2^)			
Overall	43.73	45.54	
Protein	38.94	38.69	
Ligands	50.17	53.64	
Solvent	42.09	41.76	

Values in parentheses are for the outer shell.

Consistent with previously determined Usb1 structures, KmUsb1 retains the canonical 2H fold, with two conserved HxS motifs buried in the central cleft between the transit and terminal lobes in a very narrow channel that is just wide enough to accommodate single stranded, but not double stranded RNA (Supplementary Figure S2C). In addition, two glycerol molecules are bound in the catalytic pocket as high-occupancy ligands (calculated values are between 0.87 and 1.0), where one of the expected catalysts (His211) forms a hydrogen bond to a hydroxyl group of a glycerol molecule (Supplementary Figure S2B).

### Coordination of uridine 5′-monophosphate in the active site

**Figure 2.**
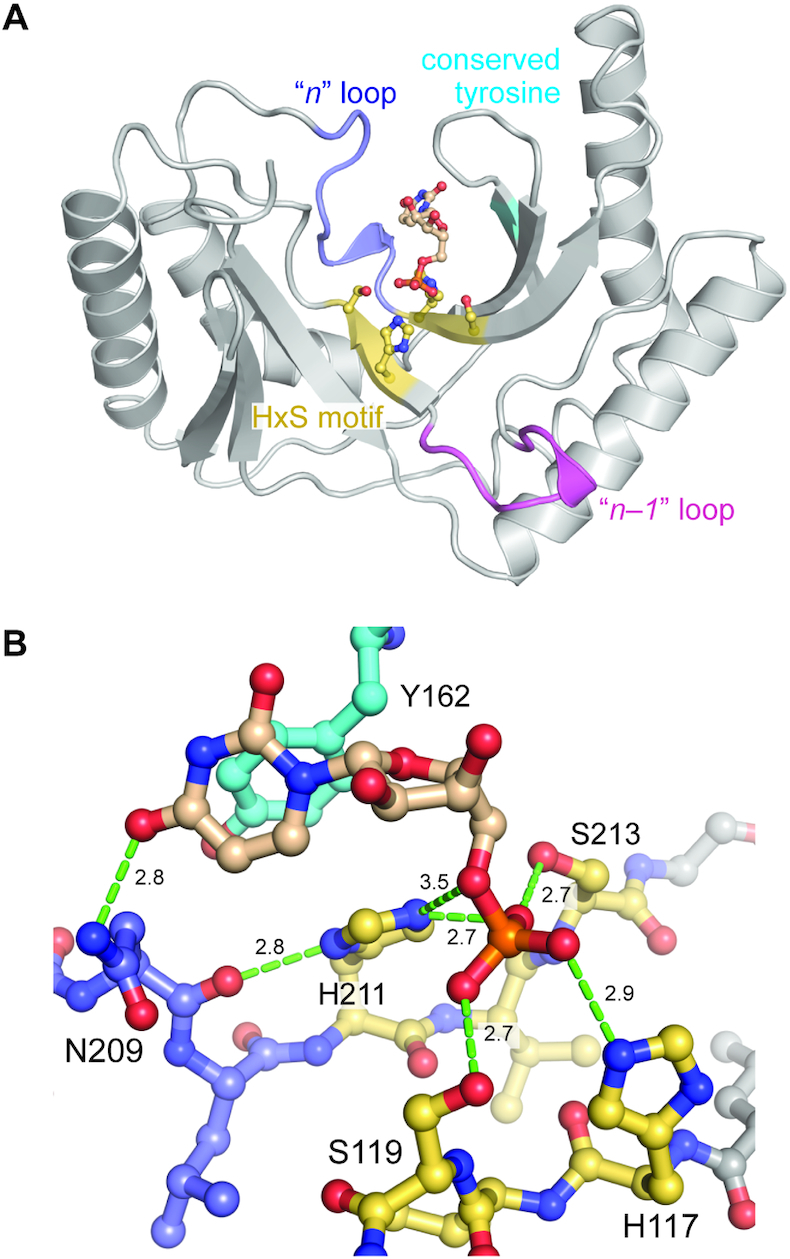
The crystal structure of *K. marxianu*s Usb1. (**A**) Overview of the co-crystal structure of *K. marxianus* Usb1 complexed with 5′-UMP. The catalytic core of Usb1 orthologs is characterized by the symmetrical HxS motif (yellow), conserved tyrosine (cyan), ‘*n* – 1’ (magenta) and ‘*n*’ loops (purple). (**B**) Substrate mimic 5′-UMP bound in the ‘*n*’ pocket. Hydrogen bonds are depicted by green dotted lines and measured in Ångströms.

Based on the HsUsb1 co-crystal structure (14), we hypothesized that uridine 5′-monophosphate (5′-UMP) would act as a substrate analog by occupying the terminal nucleotide binding pocket (‘*n* pocket’), with its ‘scissile’ phosphate positioned in the active site. We therefore soaked KmUsb1 crystals in a solution containing UMP and PEG 400 at 4°C overnight to remove competing glycerol molecules in the active site (Supplementary Figure S2A and B). The resulting co-crystal structure of KmUsb1 bound to 5′-UMP was determined at a maximum resolution of 1.84 Å (Figure 2; Supplementary Figure S8; Table 1). As hypothesized, the 5′-UMP substrate analog is bound in the *n* pocket, with its phosphate moiety positioned between the symmetrical HxS motif of the active site (Figure 2B; Supplementary Figure S8). The protein structure changes little upon binding the substrate analog, with an r.m.s.d. of 0.3 Å for all alpha-carbon atoms between the free and UMP-bound structures. H211 is positioned 3.5 Å away from the 5′ oxygen of the phosphate moiety of UMP and therefore is likely to function as the catalytic acid during the first cleavage step, analogous to previous structural and mechanistic data for HsUsb1 (5). We note that in the KmUsb1 structure, H211 is not perfectly positioned for catalysis and forms a hydrogen bond to a non-bridging oxygen of 5′-UMP (Figure 2B). The O4 oxygen of UMP forms a hydrogen bond to the sidechain nitrogen of Asn209 (Figure 2B). Tyr162 forms a T-shaped stacking interaction (40) with the uracil nucleobase and also stacks against the C-terminal catalytic histidine (Figure 2B).

**Figure 3.**
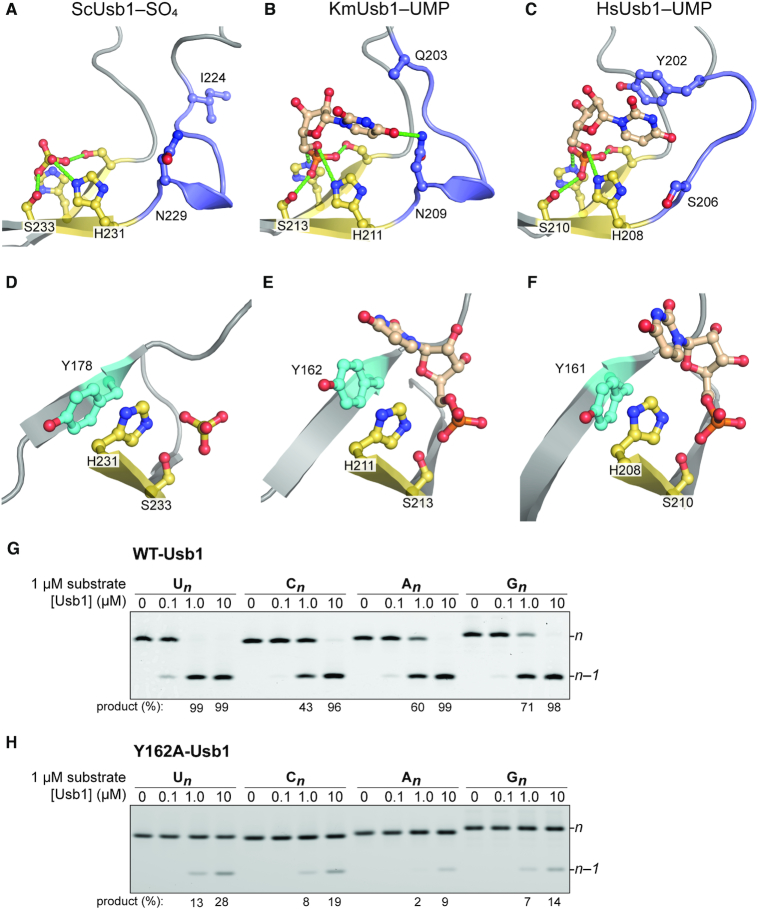
Comparison of the active-site architectures among Usb1 orthologs. (A–C) The terminal nucleobase coordination by the ‘*n*’ loop. (**A**) ScUsb1–SO_4_ (PDB 5UQJ). (**B**) KmUsb1–5′UMP (PDB 6PGL). (**C**) HsUsb1–5′UMP (PDB 5V1M). The *n* loop architectures adjacent to the C-terminal HxS motif are highlighted as in Figure 2A. Hydrogen bonds are depicted as in Figure 2B. Representative residues are rendered as ball and stick. (bottom rows, **D**–**F**) Close-up views of the conserved tyrosine in the *n* binding pockets of Usb1 orthologs. The conserved tyrosine stacks on the C-terminal catalyst and the *n* nucleobase, the interaction of which facilitates catalysis. (**G**) The terminal nucleotide preference of *K. marxianus* Usb1. Product (%) in the presence of 1.0 and 10 μM Usb1 is indicated below. (**H**) Effect of mutating Tyr162 to alanine on Usb1 catalysis. Assays were performed in the same conditions as for wild-type Usb1. Product (%) in the presence of Usb1 (1–10 μM) is indicated below.

The overall *K*. *marxianus*, *S*. *cerevisiae* and human Usb1 structures are highly homologous, although KmUsb1 more closely resembles ScUsb1 (the r.m.s.d. values are 3.1 Å for 182 aligned alpha carbon residues between KmUsb1 versus ScUsb1, and 4.1 Å for 159 aligned alpha carbon residues between KmUsb1 versus HsUsb1). The two HxS motifs in the active sites are all in nearly identical conformations, regardless of the presence of a bound ligand (Supplementary Figure S9: the all-atom r.m.s.d. values of the histidines and serines in the HxS motifs are within 0.08–0.40). The active site of KmUsb1 is similar to ScUsb1 as both have a 3_10_-helix loop architecture in the *n* nucleotide binding pocket (residues 207–209 in KmUsb1) which is absent in HsUsb1 (Figure 3A–C; Supplementary Figure S6B). An asparagine residue (Asn209), interacts with the nucleobase via a hydrogen bond to the uracil O4 (Figures 2B and 3B).

We measured the extent of processing for substrate RNAs that terminate in four different nucleotides. KmUsb1 favors RNA substrates with a terminal uridine, has weaker activity towards guanosine followed by adenosine and cytidine (U>G>A>C) (Figure 3G). The nucleotide preference of *K*. *marxianus* differs from the human ortholog, which is more reactive towards terminal adenosines (A>U>G>C) (5).

Previously determined co-crystal structures of 2H proteins bound to RNA also have an aromatic residue in the active site that stacks with a nucleobase (4–6,9,10,14). In HsUsb1, a stacking interaction between Tyr202 and the terminal nucleotide is important for Usb1 catalysis (Figure 3C) (5). However, KmUsb1 and ScUsb1 lack a corresponding aromatic residue. In KmUsb1, there is a glutamine instead of tyrosine (Gln203), the side chain of which is not visible and is therefore presumed disordered in the co-crystal structure (Figure 3B). Other notable differences include the 5′-UMP sugar pucker, which is C2′-endo and C3′-endo in the KmUsb1–5′-UMP and HsUsb1–5′-UMP structures, respectively (Figure 3B, C, E and F). Additionally, the nucleobase orientation differs slightly between the two structures, owing in part to the different modes of recognition involving H-bonding vs. stacking (Figure 3B, C, E and F). These differences in coordinating the *n* nucleobase likely contribute to nucleotide specificity among Usb1 orthologs.

We constructed a structure-based sequence alignment and identified three residues that are fully conserved among Usb1 proteins in addition to the catalytic residues (Supplementary Figure S6B). A universally conserved tyrosine residue stacks against the C-terminal catalytic histidine and nucleobase in the nucleotide bound structures (Figure 3D–F). We tested the functional importance of this tyrosine in KmUsb1 by mutating to alanine. The resulting Y162A mutant shows a significant reduction in catalytic activity, therefore confirming that this highly conserved tyrosine is important for catalysis (Figure 3H).

### The *n* – 1 nucleotide loop architecture

**Figure 4.**
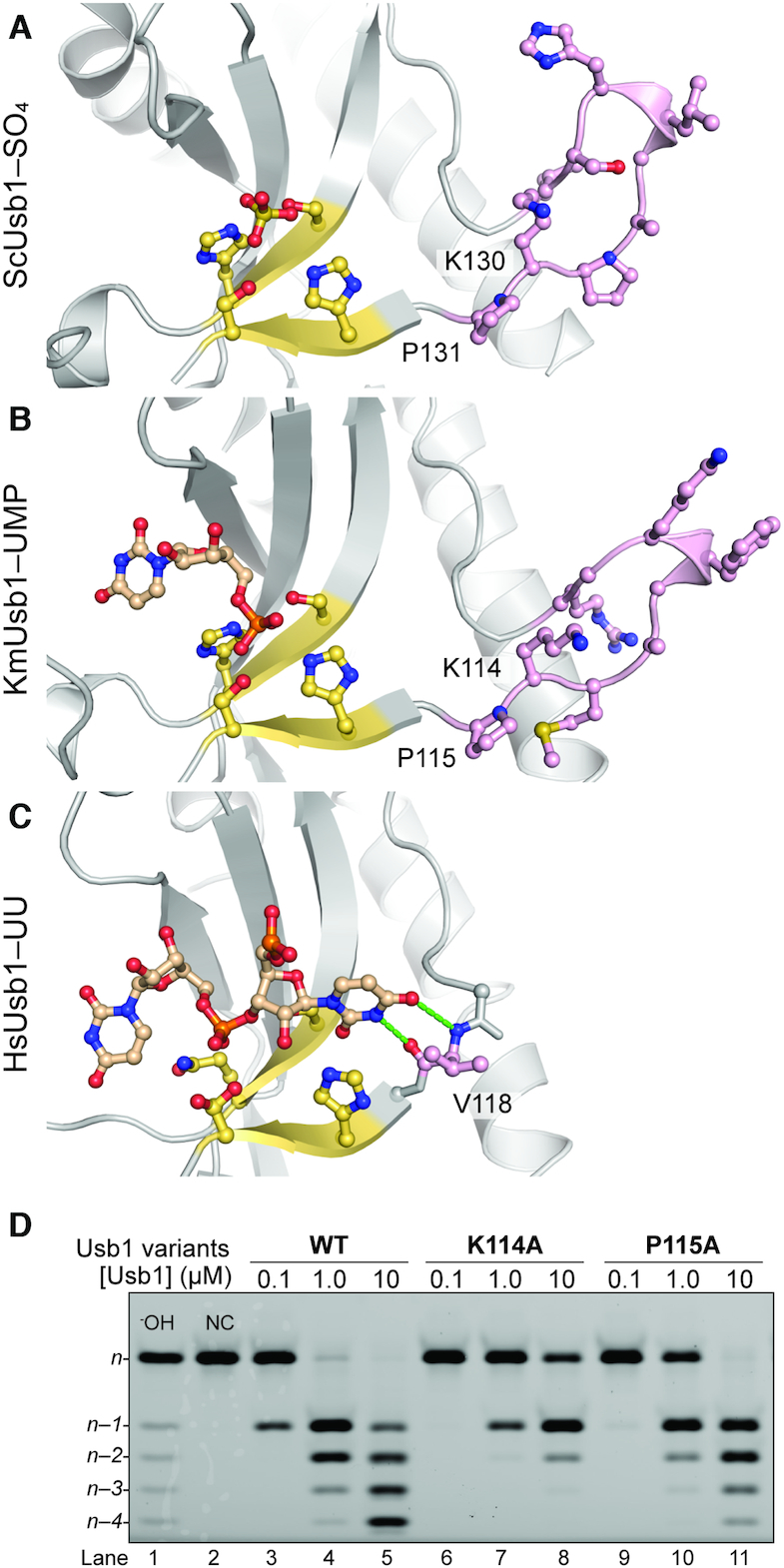
Yeast-specific loop insertion at the *n* – 1 pocket. (A–C) Close-up views of the ‘*n* – 1’ binding pocket. The extended loops of *K. marxianu*s and *S. cerevisiae* Usb1 are adjacent to the N-terminal catalytic motif and contain nine amino acids, whereas human Usb1 has only a single amino acid (Val118) which hydrogen bonds to the *n* – 1 uracil (green dotted lines) (5). The *n* – 1 loop and the HxS motifs are highlighted and depicted as in Figure 2. (**A**) ScUsb1–SO_4_ (PDB: 5UQJ). (**B**) KmUsb1–5′UMP (PDB: 6PGL). (**C**) HsUsb1–UU (PDB: 6D30). (**D**) Importance of the yeast-specific *n* – 1 loop on Usb1 catalysis. Alanine substitution of lysine and proline in the *n* – 1 loop has a detrimental effect on Usb1 catalysis.

A major difference in the Usb1 structures corresponds to the *n–1* binding pocket (‘*n* – 1 loop’) (Figure 4A–C). In the structures of HsUsb1 bound to UUUU and UUUA (5), the *n–1* nucleobase is coordinated via hydrogen bonds with the backbone of Val118 in the *n* – 1 loop (Figure 4C; Supplementary Figure S6B). In contrast, this loop is much larger in both KmUsb1 and ScUsb1, with an overall positive charge and includes a 3_10_-helix structure (Figure 4A and B; Supplementary Figures S6B and S10). The yeast-specific architecture suggests that the coordination mechanism of the *n* – 1 nucleotide is similar in both organisms, and differs from human Usb1. A lysine and proline residue are conserved in yeast enzymes (Lys114 and Pro115 in KmUsb1: Figure 4A and B; Supplementary Figure S6B). We tested to what extent these residues affect Usb1 catalysis using alanine substitutions. These mutations resulted in slower kinetics compared to wild type Usb1 (Figure 4D), with the greatest defect observed for K114A (Figure 4D, lanes 7 and 8). We hypothesize that the lysine is involved in contacting the U6 substrate and the proline is important for forming the *n* – 1 loop architecture. Unlike the shorter *n–1* loop in HsUsb1 that can accommodate only the penultimate nucleotide (Figure 4C), the extended loop in yeast may be capable of interacting with additional nucleotides (Figure 4A and B). We therefore wondered if the fungal *n* – 1 loop structure is important for the second step CPDase activity shared by ScUsb1 and KmUsb1.

**Figure 5.**
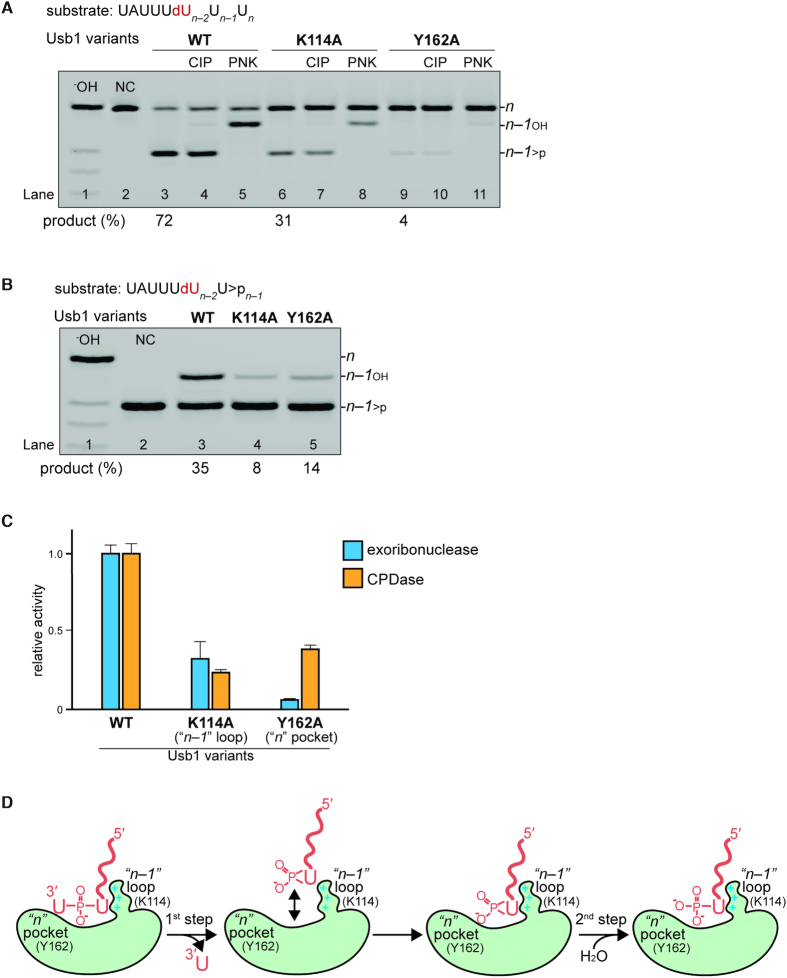
Effect of mutating the residues in the *n* – 1 and *n* binding pockets on exoribonuclease cleavage and CPDase activities. (**A**) Exoribonuclease activities of wild type (WT: lane3), K114A (lane 6) and Y162A (lane 9) proteins. The 3′ end cyclic phosphate of products was confirmed by CIP and PNK (WT: lanes 4 and 5; K114A: lanes 6 and 7; Y162A: lanes 10 and 11). (**B**) CPDase activities of WT (lane3), K114A (lane 4) and Y162A (lane 5) proteins. All products were treated with CIP to measure the ratio of the terminal mono phosphate to cyclic phosphate. (**C**) Comparison of wild type Usb1 activity with mutants. Error bars indicate the standard deviation in triplicate measurements. (**D**) Proposed mechanism for the two-step cleavage reaction. The terminal two nucleotides of U6 snRNA are coordinated by interacting with the *n* – 1 and *n* binding pockets where Y162 has a greater effect on exoribonuclease activity. Subsequent product RNA with the terminal cyclic phosphate is coordinated by the *n* – 1 loop of Usb1 enzymes harboring CPDase activity, promoting hydrolysis of the cyclic phosphate.

We independently measured the first (exoribonuclease) and second (CPDase) steps for the wild type and mutant KmUsb1. The mutations were made to the *n* pocket (Y162A: Figure 3E) or to the *n* – 1 pocket (K114A: Figure 4B). By using a single-cleavage substrate and a relatively short incubation time (10 min), the exoribonuclease step can be isolated and the product contains almost exclusively a 2′,3′-cyclic phosphate rather than a 3′ phosphate, as confirmed by CIP and PNK treatment (Figure 5A, lanes 4 and 5). Both mutants exhibit a significant defect in exoribonuclease activity relative to wild type Usb1. Y162A has a greater defect than K114A, indicating the importance of stacking on the terminal nucleobase during the first catalytic step (Figures 3E, H and 5A). We also compared second step CPDase activity by utilizing a substrate terminating in a 2′,3′-cyclic phosphate and containing a 2′-deoxy modification at the *n* – 2, position to prevent further processing (Figure 5B; Supplementary Figure S5). In this assay, the defects are reversed: K114A hydrolyzes the cyclic phosphate less well than the Y162A mutant (Figure 5B and C). These results can be explained by the model depicted in Figure 5D. During the first step of catalysis, the terminal *n* nucleotide occupies the *n* pocket, corresponding to the UMP-bound crystal structures (Figure 3B and E). After the first step in catalysis, the cleaved terminal *n* nucleotide dissociates from the *n* pocket. Interactions between the cyclic phosphate product and the *n* – 1 loop promote second step CPDase activity (Figure 5D).

## DISCUSSION

Although Usb1 enzymes have significantly diverged throughout evolution, KmUsb1 is more closely related to ScUsb1 in both sequence and structure than it is to HsUsb1. It is therefore interesting that KmUsb1 can process the 3′ end of U6 into progressively shorter products, similar to HsUsb1 but not ScUsb1. Progressive processing of U6 snRNA by Usb1 requires the presence of an RNA end with either a 3′ hydroxyl or a 2′,3′ cyclic phosphate (11,14). In contrast, a 3′ phosphate substrate cannot be processed by KmUsb1, ScUsb1 or HsUsb1 (11,14). In the case of KmUsb1, a 2′,3′ cyclic phosphate on the substrate persists due to weak CPDase activity, whereas in HsUsb1 it persists due to complete lack of detectable CPDase activity (5,14). In contrast, ScUsb1 has efficient CPDase activity resulting in a two-step catalytic mechanism that produces a 3′ phosphate which halts the enzyme from further processing U6 snRNA, leading to cleavage of a single nucleotide, predominately (14). In general, there appear to be several factors that redundantly prevent U6 snRNA from being over-processed by Usb1. First, Usb1 is an intrinsically inefficient enzyme, ∼10^6^-fold slower than RNase A (5). Additionally, the secondary structure of U6 snRNA and/or interactions with other spliceosomal components are likely to further block Usb1 from over-processing (5).

Although KmUsb1 has weak CPDase activity, its ability to hydrolyze cyclic phosphates into 3′ monophosphates is consistent with our phylogenetic prediction based on the C-terminal sequences of Lsm8 (Supplementary Figure S1) (25). These data strongly suggest a co-evolutionary relationship between Usb1 and Lsm8. Our *in vitro* assays reveal a distributive processing mechanism in which the cyclic phosphate product can dissociate after the first catalytic step, and rebinds for hydrolysis into the 3′ phosphate (Figures 1C and 5D). In our model, the cyclic phosphate-containing RNA substrate binds the active site through interactions involving the upstream nucleotides and the *n* – 1 loop (Figure 5D). In such a scenario, the two catalytic histidines would switch catalytic roles during cyclic phosphate hydrolysis (Figure 1A), as suggested for other CPDase enzymes (41). The second catalytic step for KmUsb1 is clearly inefficient for the isolated enzyme and substrate *in vitro*, as evidenced by the accumulation of cyclic phosphate intermediates.

RNase A also accumulates cyclic phosphate intermediates due to a second catalytic step that is slower than the first step (42). We hypothesize that hydrolysis of the cyclic phosphate may be more efficient *in vivo*, especially if Usb1 is actively recruited to U6 snRNA through protein-protein interactions. In support of this hypothesis, high-throughput studies have identified many potential interaction partners for Usb1 (43–46). Some of these potential partners include known spliceosomal proteins. Consistent with this observation, it has been previously observed that mammalian U6 snRNA associated with purified spliceosomes is shortened during splicing (47). In *K. marxianus*, recruitment of Usb1 to U6 snRNA is also likely required to promote cyclic phosphate hydrolysis due to the poor catalytic efficiency observed here.

There is a striking structural similarity between the *n* – 1 loops of ScUsb1 and KmUsb1 (Figure 4A and B). This loop is proximal to where the RNA chain would extend away from the enzyme and is likely to interact with U6. Indeed, the HsUsb1 *n* – 1 loop interacts with the *n* – 1 nucleotide through main chain interactions (Figure 4C), even though the loop itself is only a single amino acid (Supplementary Figure S6B). It is also striking that the yeast *n* – 1 loop is rich in positively charged amino acids that may assist in positioning U6 snRNA into the active site of the enzyme (Figure 5D; Supplementary Figure S10A and B). It is therefore likely that this yeast-specific loop architecture evolved to assist in substrate binding, particularly for CPDase activity (Figure 5D). We hypothesize that the Usb1 CPDase activity may have evolved in yeast to inhibit over-processing of U6 snRNA, whereas other organisms such as humans have evolved orthogonal terminal uridylyl transferases that can extend and potentially ‘repair’ over-processed U6 snRNA (48).

## DATA AVAILABILITY

Coordinates and structure factors have been deposited in the Protein Data Bank with accession codes 6PFQ and 6PGL.

## Supplementary Material

gkz1177_Supplemental_File

## References

[1] Mazumder R., Iyer L.M., Vasudevan S., Aravind L. Detection of novel members, structure-function analysis and evolutionary classification of the 2H phosphoesterase superfamily. Nucleic Acids Res. 2002; 30:5229–5243. 12466548 10.1093/nar/gkf645 PMC137960

[2] Arn E.A., Abelson J.N. The 2′-5′ RNA ligase of Escherichia coli. Purification, cloning, and genomic disruption. J. Biol. Chem. 1996; 271:31145–31153. 8940112 10.1074/jbc.271.49.31145

[3] Hofmann A., Zdanov A., Genschik P., Ruvinov S., Filipowicz W., Wlodawer A. Structure and mechanism of activity of the cyclic phosphodiesterase of Appr>p, a product of the tRNA splicing reaction. EMBO J. 2000; 19:6207–6217. 11080166 10.1093/emboj/19.22.6207 PMC305825

[4] Remus B.S., Jacewicz A., Shuman S. Structure and mechanism of E. coli RNA 2′,3′-cyclic phosphodiesterase. RNA. 2014; 20:1697–1705. 25239919 10.1261/rna.046797.114 PMC4201822

[5] Nomura Y., Roston D., Montemayor E.J., Cui Q., Butcher S.E. Structural and mechanistic basis for preferential deadenylation of U6 snRNA by Usb1. Nucleic Acids Res. 2018; 46:11488–11501. 30215753 10.1093/nar/gky812 PMC6265477

[6] Hofmann A., Grella M., Botos I., Filipowicz W., Wlodawer A. Crystal structures of the semireduced and inhibitor-bound forms of cyclic nucleotide phosphodiesterase from Arabidopsis thaliana. J. Biol. Chem. 2002; 277:1419–1425. 11694509 10.1074/jbc.M107889200

[7] Kato M., Shirouzu M., Terada T., Yamaguchi H., Murayama K., Sakai H., Kuramitsu S., Yokoyama S. Crystal structure of the 2′-5′ RNA ligase from Thermus thermophilus HB8. J. Mol. Biol. 2003; 329:903–911. 12798681 10.1016/s0022-2836(03)00448-0

[8] Gao Y.G., Yao M., Okada A., Tanaka I. The structure of Pyrococcus horikoshii 2′-5′ RNA ligase at 1.94 A resolution reveals a possible open form with a wider active-site cleft. Acta Crystallogr. F, Struct. Biol. Crystall. Commun. 2006; 62:1196–1200. 10.1107/S1744309106046616 PMC2225383 17142895

[9] Gold M.G., Smith F.D., Scott J.D., Barford D. AKAP18 contains a phosphoesterase domain that binds AMP. J. Mol. Biol. 2008; 375:1329–1343. 18082768 10.1016/j.jmb.2007.11.037 PMC3188456

[10] Myllykoski M., Raasakka A., Han H., Kursula P. Myelin 2′,3′-cyclic nucleotide 3′-phosphodiesterase: active-site ligand binding and molecular conformation. PLoS One. 2012; 7:e32336. 22393399 10.1371/journal.pone.0032336 PMC3290555

[11] Hilcenko C., Simpson P.J., Finch A.J., Bowler F.R., Churcher M.J., Jin L., Packman L.C., Shlien A., Campbell P., Kirwan M. et al. Aberrant 3′ oligoadenylation of spliceosomal U6 small nuclear RNA in poikiloderma with neutropenia. Blood. 2013; 121:1028–1038. 23190533 10.1182/blood-2012-10-461491

[12] Myllykoski M., Raasakka A., Lehtimaki M., Han H., Kursula I., Kursula P. Crystallographic analysis of the reaction cycle of 2′,3′-cyclic nucleotide 3′-phosphodiesterase, a unique member of the 2H phosphoesterase family. J. Mol. Biol. 2013; 425:4307–4322. 23831225 10.1016/j.jmb.2013.06.012 PMC7094350

[13] Raasakka A., Myllykoski M., Laulumaa S., Lehtimaki M., Hartlein M., Moulin M., Kursula I., Kursula P. Determinants of ligand binding and catalytic activity in the myelin enzyme 2′,3′-cyclic nucleotide 3′-phosphodiesterase. Sci. Rep. 2015; 5:16520. 26563764 10.1038/srep16520 PMC4643303

[14] Didychuk A.L., Montemayor E.J., Carrocci T.J., DeLaitsch A.T., Lucarelli S.E., Westler W.M., Brow D.A., Hoskins A.A., Butcher S.E. Usb1 controls U6 snRNP assembly through evolutionarily divergent cyclic phosphodiesterase activities. Nat. Commun. 2017; 8:497. 28887445 10.1038/s41467-017-00484-w PMC5591277

[15] Myllykoski M., Kursula P. Structural aspects of nucleotide ligand binding by a bacterial 2H phosphoesterase. PLoS One. 2017; 12:e0170355. 28141848 10.1371/journal.pone.0170355 PMC5283653

[16] Volpi L., Roversi G., Colombo E.A., Leijsten N., Concolino D., Calabria A., Mencarelli M.A., Fimiani M., Macciardi F., Pfundt R. et al. Targeted next-generation sequencing appoints c16orf57 as clericuzio-type poikiloderma with neutropenia gene. Am. J. Hum. Genet. 2010; 86:72–76. 20004881 10.1016/j.ajhg.2009.11.014 PMC2801743

[17] Mroczek S., Krwawicz J., Kutner J., Lazniewski M., Kucinski I., Ginalski K., Dziembowski A. C16orf57, a gene mutated in poikiloderma with neutropenia, encodes a putative phosphodiesterase responsible for the U6 snRNA 3′ end modification. Genes Dev. 2012; 26:1911–1925. 22899009 10.1101/gad.193169.112 PMC3435495

[18] Shchepachev V., Wischnewski H., Missiaglia E., Soneson C., Azzalin C.M. Mpn1, mutated in poikiloderma with neutropenia protein 1, is a conserved 3′-to-5′ RNA exonuclease processing U6 small nuclear RNA. Cell Rep. 2012; 2:855–865. 23022480 10.1016/j.celrep.2012.08.031

[19] Shchepachev V., Wischnewski H., Soneson C., Arnold A.W., Azzalin C.M. Human Mpn1 promotes post-transcriptional processing and stability of U6atac. FEBS Lett. 2015; 589:2417–2423. 26213367 10.1016/j.febslet.2015.06.046

[20] Fica S.M., Nagai K. Cryo-electron microscopy snapshots of the spliceosome: structural insights into a dynamic ribonucleoprotein machine. Nat. Struct. Mol. Biol. 2017; 24:791–799. 28981077 10.1038/nsmb.3463 PMC6386135

[21] Didychuk A.L., Butcher S.E., Brow D.A. The life of U6 small nuclear RNA, from cradle to grave. RNA. 2018; 24:437–460. 29367453 10.1261/rna.065136.117 PMC5855946

[22] Stefano J.E. Purified lupus antigen La recognizes an oligouridylate stretch common to the 3′ termini of RNA polymerase III transcripts. Cell. 1984; 36:145–154. 6607117 10.1016/0092-8674(84)90083-7

[23] Kunkel G.R., Maser R.L., Calvet J.P., Pederson T. U6 small nuclear RNA is transcribed by RNA polymerase III. Proc. Natl. Acad. Sci. U.S.A. 1986; 83:8575–8579. 3464970 10.1073/pnas.83.22.8575 PMC386973

[24] Licht K., Medenbach J., Luhrmann R., Kambach C., Bindereif A. 3′-cyclic phosphorylation of U6 snRNA leads to recruitment of recycling factor p110 through LSm proteins. RNA. 2008; 14:1532–1538. 18567812 10.1261/rna.1129608 PMC2491463

[25] Montemayor E.J., Didychuk A.L., Yake A.D., Sidhu G.K., Brow D.A., Butcher S.E. Architecture of the U6 snRNP reveals specific recognition of 3′-end processed U6 snRNA. Nat. Commun. 2018; 9:1749. 29717126 10.1038/s41467-018-04145-4 PMC5931518

[26] Wolfe K.H., Shields D.C. Molecular evidence for an ancient duplication of the entire yeast genome. Nature. 1997; 387:708–713. 9192896 10.1038/42711

[27] Kapust R.B., Tozser J., Fox J.D., Anderson D.E., Cherry S., Copeland T.D., Waugh D.S. Tobacco etch virus protease: mechanism of autolysis and rational design of stable mutants with wild-type catalytic proficiency. Protein Eng. 2001; 14:993–1000. 11809930 10.1093/protein/14.12.993

[28] Fox J.D., Waugh D.S. Maltose-binding protein as a solubility enhancer. Methods Mol. Biol. 2003; 205:99–117. 12491882 10.1385/1-59259-301-1:99

[29] Garman E., Murray J.W. Heavy-atom derivatization. Acta Crystallogr. D, Biol. Crystallogr. 2003; 59:1903–1913. 14573944 10.1107/s0907444903012794

[30] Kabsch W. XDS. Acta Crystallogr. D, Biol. Crystallogr. 2010; 66:125–132. 20124692 10.1107/S0907444909047337 PMC2815665

[31] Evans P.R., Murshudov G.N. How good are my data and what is the resolution?. Acta Crystallogra. D, Biol. Crystallogr. 2013; 69:1204–1214. 10.1107/S0907444913000061 PMC3689523 23793146

[32] Sheldrick G.M. A short history of SHELX. Acta Crystallogr. A, Found. Crystallogr. 2008; 64:112–122. 10.1107/S0108767307043930 18156677

[33] Pape T., Schneider T.R. HKL2MAP: a graphical user interface for macromolecular phasing with SHELX programs. J. Appl. Crystallogr. 2004; 37:843–844.

[34] Adams P.D., Afonine P.V., Bunkoczi G., Chen V.B., Davis I.W., Echols N., Headd J.J., Hung L.W., Kapral G.J., Grosse-Kunstleve R.W. et al. PHENIX: a comprehensive Python-based system for macromolecular structure solution. Acta Crystallogr. D, Biol. Crystallogr. 2010; 66:213–221. 20124702 10.1107/S0907444909052925 PMC2815670

[35] Afonine P.V., Grosse-Kunstleve R.W., Echols N., Headd J.J., Moriarty N.W., Mustyakimov M., Terwilliger T.C., Urzhumtsev A., Zwart P.H., Adams P.D. Towards automated crystallographic structure refinement with phenix.refine. Acta Crystallogr. D, Biol. Crystallogr. 2012; 68:352–367. 22505256 10.1107/S0907444912001308 PMC3322595

[36] Vonrhein C., Tickle I., Flensburg C., Keller P., Paciorek W., Sharff A. Advances in automated data analysis and processing within autoPROC, combined with improved characterisation, mitigation and visualisation of the anisotropy of diffraction limits using STARANISO. Found. Crystallogr. 2018; 74:a360.

[37] Emsley P., Lohkamp B., Scott W.G., Cowtan K. Features and development of Coot. Acta Crystallogr. D, Biol. Crystallogr. 2010; 66:486–501. 20383002 10.1107/S0907444910007493 PMC2852313

[38] Murshudov G.N., Vagin A.A., Dodson E.J. Refinement of macromolecular structures by the maximum-likelihood method. Acta Crystallogr. D, Biol. Crystallogr. 1997; 53:240–255. 15299926 10.1107/S0907444996012255

[39] Winn M.D., Ballard C.C., Cowtan K.D., Dodson E.J., Emsley P., Evans P.R., Keegan R.M., Krissinel E.B., Leslie A.G., McCoy A. et al. Overview of the CCP4 suite and current developments. Acta Crystallogr. D, Biol. Crystallogr. 2011; 67:235–242. 21460441 10.1107/S0907444910045749 PMC3069738

[40] McGaughey G.B., Gagne M., Rappe A.K. pi-Stacking interactions. Alive and well in proteins. J. Biol. Chem. 1998; 273:15458–15463. 9624131 10.1074/jbc.273.25.15458

[41] Raines R.T. Ribonuclease A. Chem. Rev. 1998; 98:1045–1066. 11848924 10.1021/cr960427h

[42] Thompson J.E., Venegas F.D., Raines R.T. Energetics of catalysis by ribonucleases: fate of the 2′,3′-cyclic phosphodiester intermediate. Biochemistry. 1994; 33:7408–7414. 8003506 10.1021/bi00189a047

[43] Hazbun T.R., Malmstrom L., Anderson S., Graczyk B.J., Fox B., Riffle M., Sundin B.A., Aranda J.D., McDonald W.H., Chiu C.H. et al. Assigning function to yeast proteins by integration of technologies. Mol. Cell. 2003; 12:1353–1365. 14690591 10.1016/s1097-2765(03)00476-3

[44] Gavin A.C., Aloy P., Grandi P., Krause R., Boesche M., Marzioch M., Rau C., Jensen L.J., Bastuck S., Dumpelfeld B. et al. Proteome survey reveals modularity of the yeast cell machinery. Nature. 2006; 440:631–636. 16429126 10.1038/nature04532

[45] Krogan N.J., Cagney G., Yu H., Zhong G., Guo X., Ignatchenko A., Li J., Pu S., Datta N., Tikuisis A.P. et al. Global landscape of protein complexes in the yeast Saccharomyces cerevisiae. Nature. 2006; 440:637–643. 16554755 10.1038/nature04670

[46] Yu H., Braun P., Yildirim M.A., Lemmens I., Venkatesan K., Sahalie J., Hirozane-Kishikawa T., Gebreab F., Li N., Simonis N. et al. High-quality binary protein interaction map of the yeast interactome network. Science. 2008; 322:104–110. 18719252 10.1126/science.1158684 PMC2746753

[47] Tazi J., Forne T., Jeanteur P., Cathala G., Brunel C. Mammalian U6 small nuclear RNA undergoes 3′ end modifications within the spliceosome. Mol. Cell Biol. 1993; 13:1641–1650. 8441402 10.1128/mcb.13.3.1641-1650.1993 PMC359476

[48] Trippe R., Guschina E., Hossbach M., Urlaub H., Luhrmann R., Benecke B.J. Identification, cloning, and functional analysis of the human U6 snRNA-specific terminal uridylyl transferase. RNA. 2006; 12:1494–1504. 16790842 10.1261/rna.87706 PMC1524887

