# Supplementary material for: Structural basis for the evolution of cyclic phosphodiesterase activity in the U6 snRNA exoribonuclease Usb1
Source: Nucleic Acids Res. 2019 Dec 13;48(3):1423–34. doi: 10.1093/nar/gkz1177 (PMC7026655; doi:10.1093/nar/gkz1177)
Supplement: gkz1177_Supplemental_File [file gkz1177_supplemental_file.docx]

Supplementary Data

**Structural basis for the evolution of cyclic phosphodiesterase activity in the U6 snRNA exoribonuclease Usb1**

Yuichiro Nomura, Eric J. Montemayor, Johanna M. Virta, Samuel M. Hayes and Samuel E. Butcher*

Department of Biochemistry, University of Wisconsin, Madison, Wisconsin 53706, USA

*To whom correspondence should be addressed. Tel: +1 608 263 3890; Email: [sebutcher@wisc.edu](mailto:sebutcher@wisc.edu)

**SUPPLEMENTARY MATERIALS**

**Sequences of protein-coding ORFs and oligonucleotides**

# Codon optimized *Kluyveromyces marxianus* Usb1 gene.

# Full-length Usb1 (residues 1–275):

# 5ʹ‑ATGGGTCTGGTGAGCAGCGACTATAATTCCGACTCTGACTCTGATTCAACAAACTCAGTGTCAGAAACCGGCGAAGATTCAATCAAACGGCAGTGTCTTCCCGAATTGCCTGACGACATCGTGTATAACTACAATAAGCCAGTAGTAATAGACCCCTTAAATACTCGTATGTACGTAGCACCAGTGTCCAAGAATATAGGATTTTTGTTTTTGGAGCTGCGTCTGGATTCTAAACAACAGCAGATCATGGACTTGGTCCTTAAAGGTGTTAATGCGGTCATGGACACCCATCATCGGAACTCTTTTGAACCATTGCACAGAGGCAAATTTGGGGCGATGAAGCCACTGCACGTTTCGCTTAGCGAAACGATGATGTTTGCCAATGAAAGCGAGCTGGAGGAAAAAATGGGCAGAATACGTCAAGAAATTAGAGCATTAGAGTGTAAATCAGTGCCTGTGGCTCTTAGCGGAGGCTGGTTAGTCTACGAGAATTTCGACGCAAGTCTTCAGTTCTTGGCAGTAGGGCTTAGCGAACCTGCTCGGGGCAGATTGAAGCCGGTACTTTCCATAGTCGAAAAGTATAAACCGCGCAGCCCGGTGTCACGTCAACCCGTCGGCTTAAACAACTTGCATGTGTCGTTTGGGGTGGCGCAAAATGCCTATTTACAACAAGACGAGTCTGTCTCACGGCAGCGTTTGGATAGTTTACGGAATTTAGTGGCTACCGAGGCTTCGGATCGTTTACCTCTGTTACGCGCGAACTTACAGTTTCGTTGCCACGAACTTAAAGCCAAGGTAGGAACGTCCGTCATCACTCTTCCGCTTTAATGA-3ʹ

Truncated Usb1 (residues 59–275)

5ʹ‑GTCGCCCCCGTGTCCAAAAATATAGGATTTCTTTTTCTGGAATTGCGCTTGGACTCAAAGCAACAGCAGATCATGGACCTTGTTTTAAAAGGAGTAAACGCAGTCATGGATACTCATCATAGAAATTCTTTTGAGCCACTGCATCGCGGTAAGTTTGGCGCAATGAAACCGCTGCATGTAAGTCTGTCAGAAACGATGATGTTTGCTAACGAATCTGAATTGGAGGAAAAGATGGGGAGAATACGGCAAGAAATCCGTGCCTTGGAATGTAAGTCGGTCCCTGTAGCTCTTAGCGGTGGTTGGCTGGTGTATGAGAACTTCGATGCCTCCTTACAGTTTCTGGCAGTAGGTCTGAGTGAGCCGGCGCGTGGAAGATTAAAGCCGGTTTTGTCCATAGTAGAGAAGTACAAACCTCGCTCGCCTGTTTCACGCCAACCAGTCGGCCTGAACAACCTTCACGTCAGCTTCGGTGTAGCGCAAAATGCGTATCTTCAACAGGATGAGTCAGTGAGTAGACAACGGTTAGATTCTCTTCGGAATCTTGTTGCAACCGAAGCATCAGATCGGCTGCCACTTTTGAGAGCTAACTTGCAATTTCGCTGTCACGAGCTTAAAGCTAAGGTAGGTACGTCAGTGATTACATTGCCCCTTTAA-3ʹ

K114A-Usb1 forward primer:

5ʹ-GCGCCACTGCACGTTTCGCTTAGCGAAACG-3ʹ

K114A-Usb1 reverse primer:

5ʹ-CATCGCCCCAAATTTGCCTCTGTGC-3ʹ

P115A-Usb1 forward primer:

5ʹ-GCGCTGCACGTTTCGCTTAGCGAAACGATGATG-3ʹ

P115A-Usb1 reverse primer:

5ʹ-CTTCATCGCCCCAAATTTGCCTCTGTGC-3ʹ

Y162A-Usb1 forward primer:

5ʹ-GCGGAGAATTTCGACGCAAGTCTTCAGTTC-3ʹ

Y162A-Usb1 reverse primer:

5ʹ-GACTAACCAGCCTCCGCTAAGAGCC-3ʹ

Substrate RNAs:

5ʹ-FAM-UAUUUUUU_OH_-3ʹ

5ʹ-FAM-UAUUUUdUU_OH_-3ʹ

5ʹ-FAM-UAUUUdUUU_OH_-3ʹ

5ʹ-FAM-UAUUUdUUC_OH_-3ʹ

5ʹ-FAM-UAUUUdUUA_OH_-3ʹ

5ʹ-FAM-UAUUUdUUG_OH_-3ʹ

5ʹ-FAM-UAUUUUUU_p_-3ʹ

**Supplementary Figure S1.** Prediction of the 3ʹ-phosphate modification on U6 snRNA, based on the C-terminal sequence of Lsm8. Sequence conservation is shown above the alignment for top 7 orthologs and below for the remaining, respectively. The extended C-termini of Lsm8 proteins are likely related to the 3ʹ-end non-cyclic phosphate of U6, which is produced by Usb1 enzyme with CPDase activity (1-4).

**
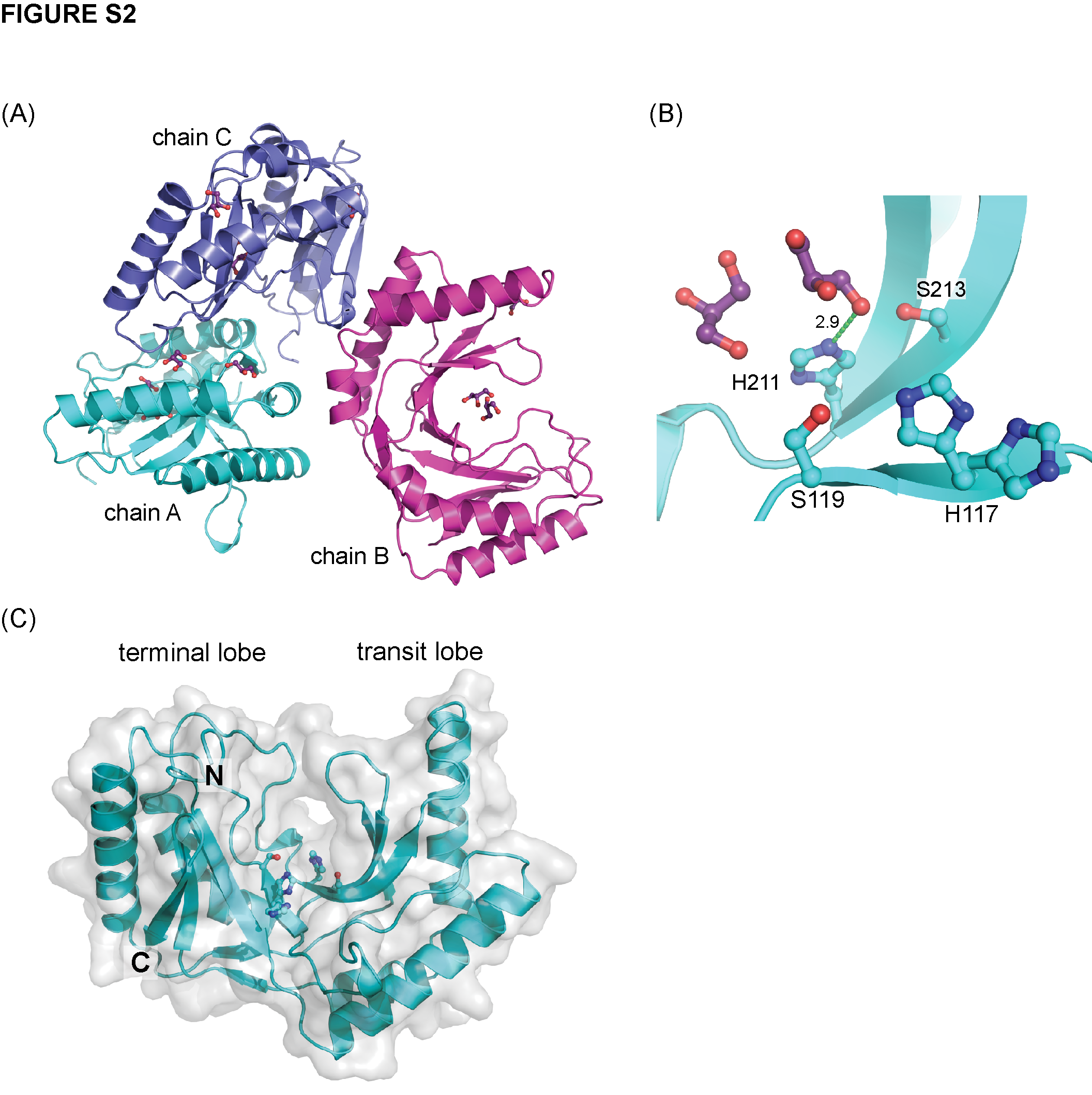
**

**Supplementary Figure S2.** The structure of *K. marxianus* Usb1 (**A**) Three subunits are present in the crystallographic asymmetric unit and are related to one another by non-crystallographic symmetry. 11 glycerol molecules (ball and stick) reside in the asymmetric unit. **(B**) Two glycerol molecules are bound in the catalytic center of all subunits, one of which forms a hydrogen bond to the C-terminal catalytic histidine H211. A hydrogen bond is depicted by a green dotted line and measured in Ångströms. (**C**) The KmUsb1 architecture is highly homologous to the other proteins within the 2H superfamily with terminal and transit lobes. The catalytic residues (HxS: ball and stick) are buried in the central cleft. The terminal lobe harbors N- and C-terminal regions.

**
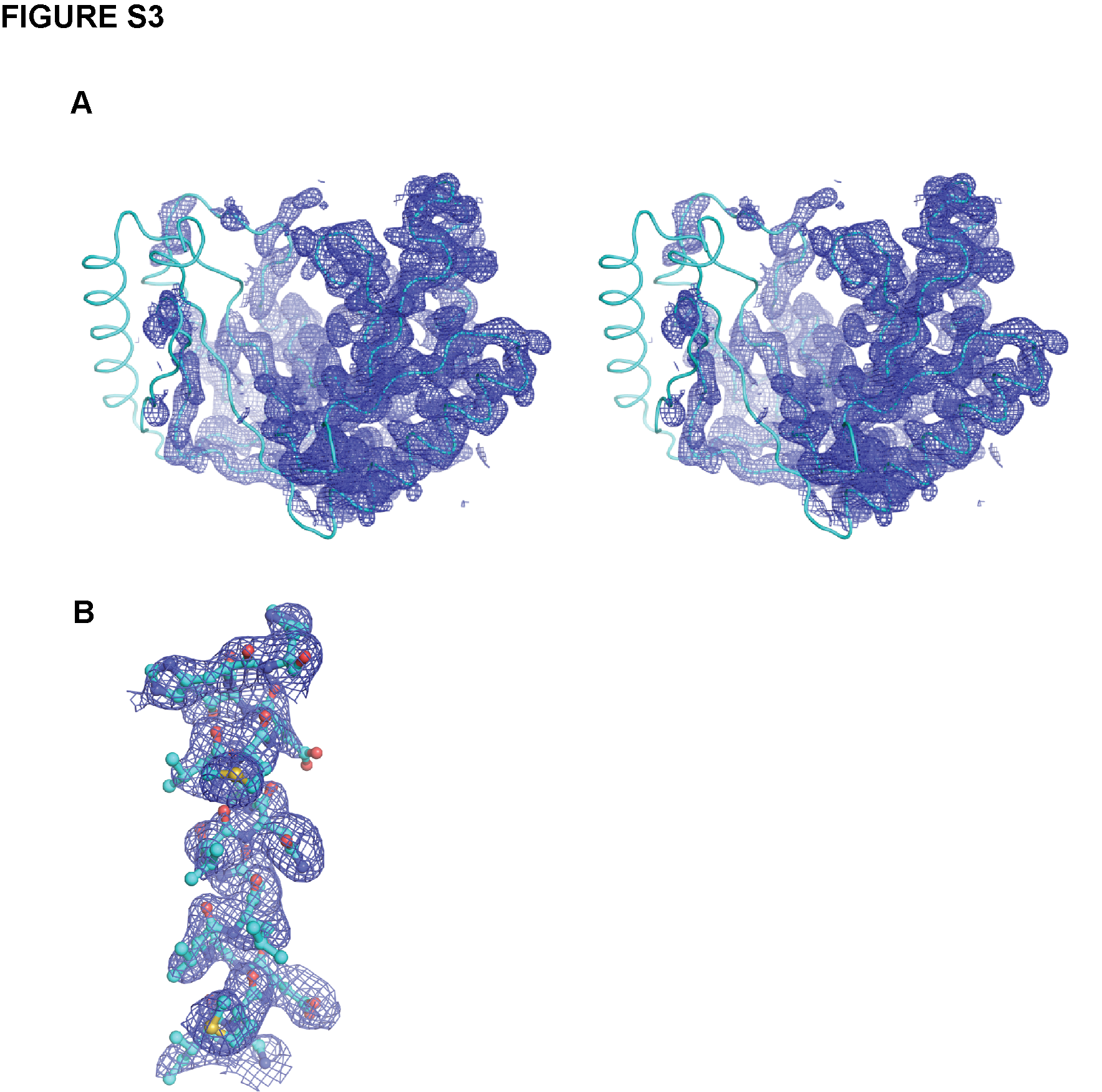
**

**Supplementary Figure S3.** Examples of the initial experimental phasing map and the final refined native structure. Electron density is contoured at 1.0 r.m.s.d. (**A**) Stereo view of the overall structure, showing that the electron density is weaker for the terminal lobe than the transit lobe. (**B**) Close-up view of a helix structure in the experimental SIRAS map.

**Supplementary Figure S4.** Optimum pH for KmUsb1 activity. KmUsb1 activity was measured at a wide range of pH using 1 µM of a single cleavage substrate (5ʹ-UAUUUdUUU-3ʹ) vs. 1 µM protein.

**Supplementary Figure S5.** Preparation of substrate RNA for measuring CPDase activity. A single-cleavage substrate with a terminal adenosine (lane 2) was shortened by HsUsb1 and the product (lane 3) was treated with either CIP (lane 4) or PNK (lane 5) to confirm the product exclusively contained a cyclic phosphate.

**
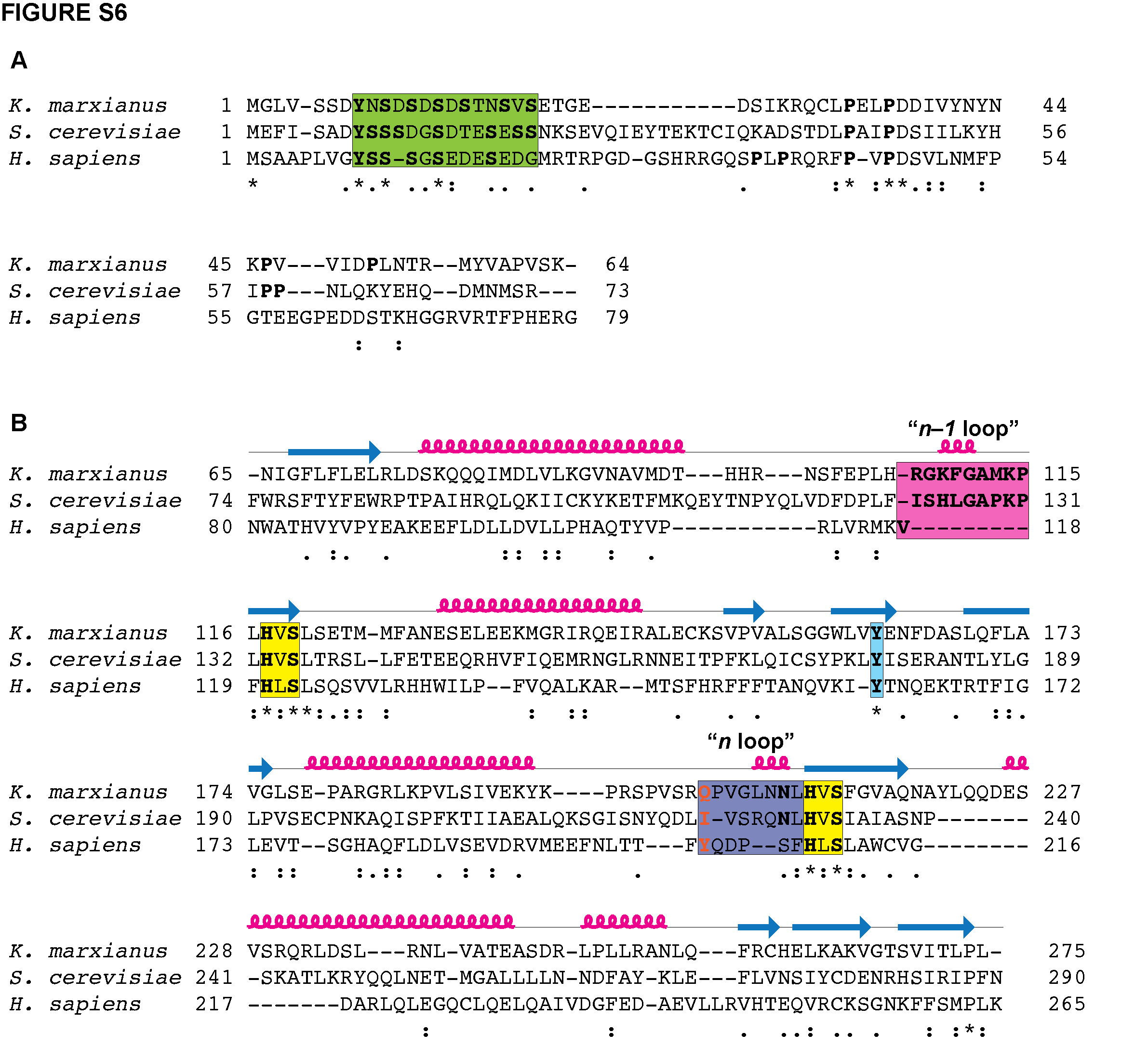
**

**Supplementary Figure S6.** Sequence comparison of Usb1 orthologs. (**A**) Sequence alignment of the N-terminal domain. The N-terminal domain is represented with a YSSS motif (green) and proline-rich region (bold). Sequence identity in the N-terminal domain: 26% between *K. marxianus* vs. *S. cerevisiae*; 19% between *K. marxianus* vs. human; 18% between *S. cerevisiae* vs. human (3). (**B**) Sequence alignment of the catalytic domain based on superimposition of Usb1 structures. The vicinal loops in the catalytic pocket and two HxS motifs are highlighted in magenta, purple and yellow, respectively (see Figures 2–4). The corresponding residues to Y202 in the human enzyme are shown in orange. There are few conserved residues in Usb1 orthologs, among which the tyrosine residue (cyan) is in position to contact with the C-terminal catalytic histidine and the terminal nucleobase (Figure 3D–F).

**Supplementary Figure S7.** Inhibition of KmUsb1 exoribonuclease activity by a 3ʹ phosphate. A minimal substrate with a *cis* diol (lanes 2–5) or with a 3ʹ monophosphate (lanes 6–9) was incubated in the presence of various concentrations of Usb1.

**
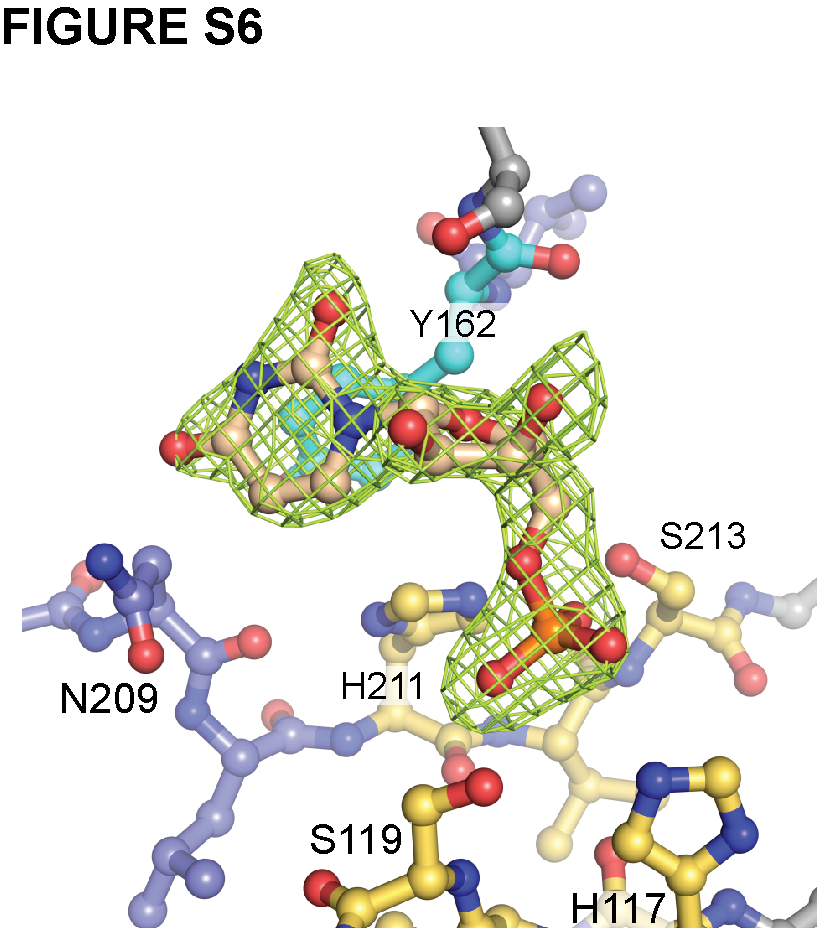
**

**Supplementary Figure S8.** Simulated annealing omit map for 5ʹ-UMP bound to the active site. The m*F*_o_–D*F*_c_ electron density map is countered at 2.0 r.m.s.d.

**
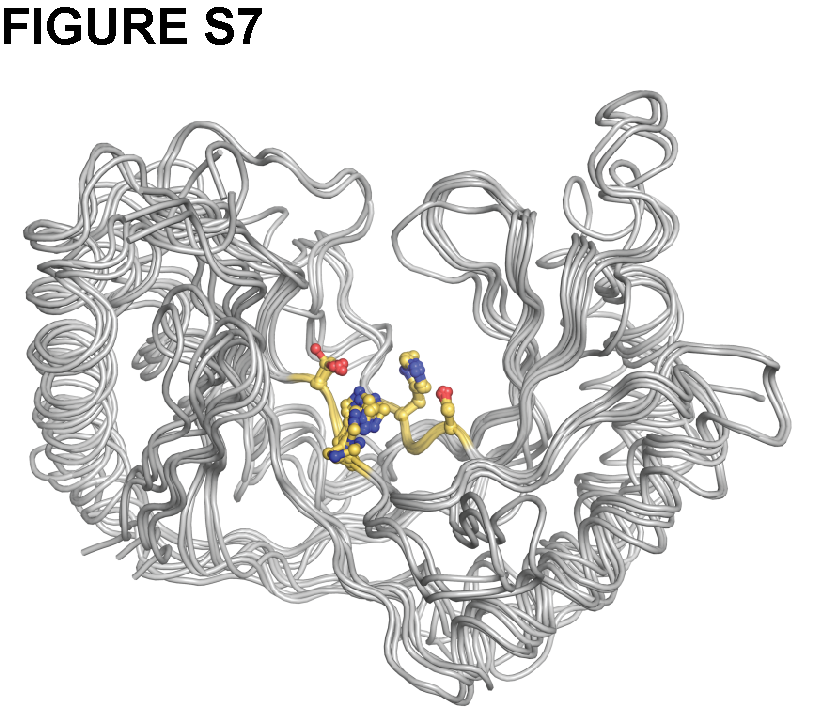
**

**Supplementary Figure S9.** Highly homologous architectures of Usb1 orthologs. Superimposition of six Usb1 structures upon two HxS motifs (yellow), from apo HsUsb1 (PDB 4H7W), HsUsb1–5ʹUMP (PDB 5V1M), HsUsb1–5ʹAMP (PDB 6D31), ScUsb1–SO_4_ (PDB 5UQJ), apo KmUsb1 (PDB 6PFQ) and KmUsb1–5ʹUMP (PDB 6PGL).

**
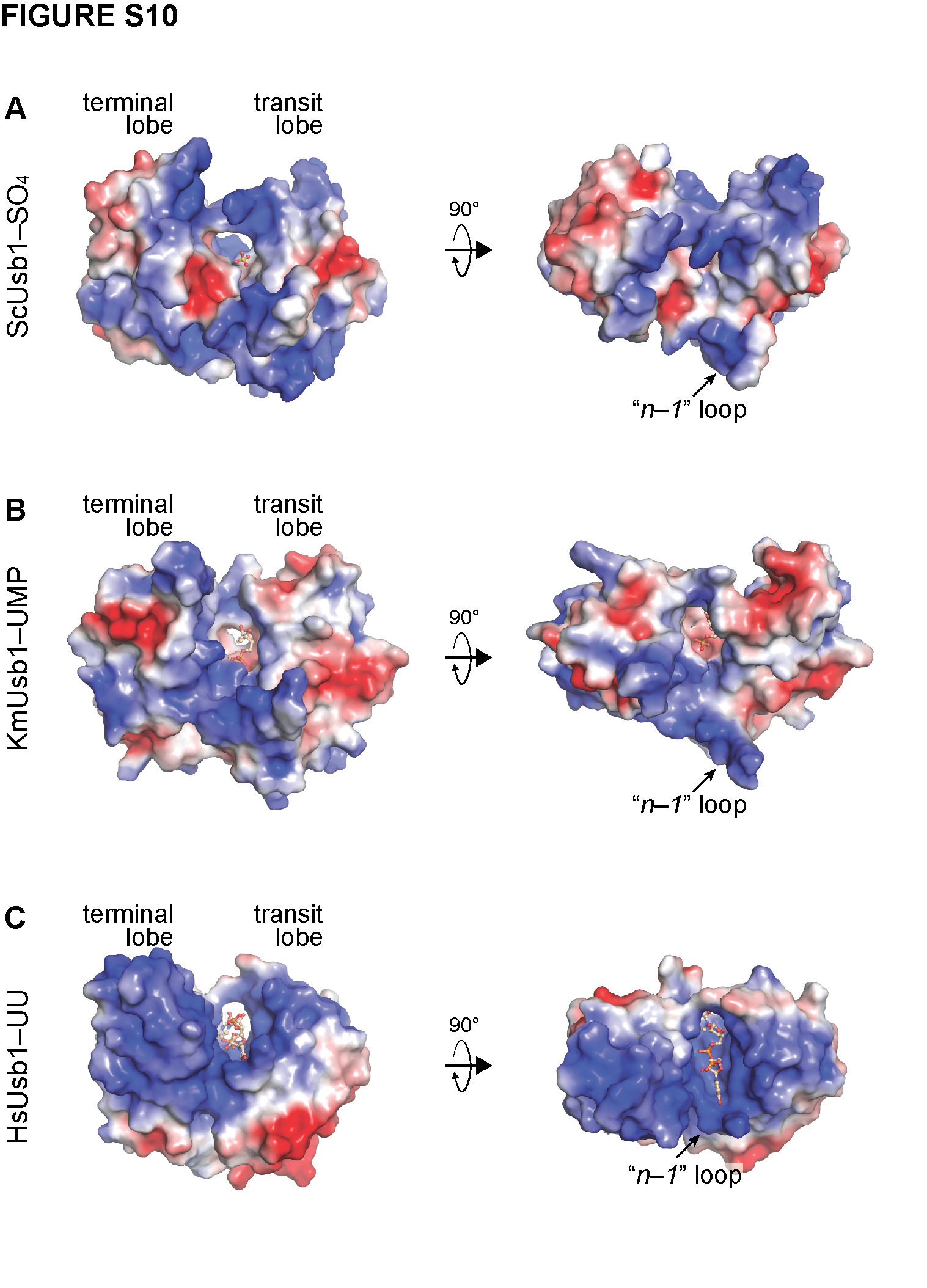
**

**Supplementary Figure S10.** The electrostatic surface of Usb1 orthologs. Surface electrostatic potentials were calculated using APBS, and contoured from +3 *kT*/*e* (blue) to –3 *kT*/*e* (red) (5). (**A**) ScUsb1–SO_4_ (PDB 5UQJ). (**B**) KmUsb1–5ʹUMP (PDB 6PGL). (**C**) HsUsb1–UU (PDB 6D30).

**REFERENCES**

1. Lund, E. and Dahlberg, J.E. (1992) Cyclic 2',3'-phosphates and nontemplated nucleotides at the 3' end of spliceosomal U6 small nuclear RNA's. *Science (New York, N.Y.)*, **255**, 327-330.

2. Mroczek, S., Krwawicz, J., Kutner, J., Lazniewski, M., Kucinski, I., Ginalski, K. and Dziembowski, A. (2012) C16orf57, a gene mutated in poikiloderma with neutropenia, encodes a putative phosphodiesterase responsible for the U6 snRNA 3' end modification. *Genes Dev*, **26**, 1911-1925.

3. Didychuk, A.L., Montemayor, E.J., Carrocci, T.J., DeLaitsch, A.T., Lucarelli, S.E., Westler, W.M., Brow, D.A., Hoskins, A.A. and Butcher, S.E. (2017) Usb1 controls U6 snRNP assembly through evolutionarily divergent cyclic phosphodiesterase activities. *Nat Commun*, **8**, 497.

4. Montemayor, E.J., Didychuk, A.L., Yake, A.D., Sidhu, G.K., Brow, D.A. and Butcher, S.E. (2018) Architecture of the U6 snRNP reveals specific recognition of 3'-end processed U6 snRNA. *Nat Commun*, **9**, 1749.

5. Baker, N.A., Sept, D., Joseph, S., Holst, M.J. and McCammon, J.A. (2001) Electrostatics of nanosystems: application to microtubules and the ribosome. *Proc Natl Acad Sci U S A*, **98**, 10037-10041.
